# Microwave Staring Correlated Imaging Based on Unsteady Aerostat Platform

**DOI:** 10.3390/s19122825

**Published:** 2019-06-24

**Authors:** Zheng Jiang, Yuanyue Guo, Jie Deng, Weidong Chen, Dongjin Wang

**Affiliations:** Key Laboratory of Electromagnetic Space Information, Chinese Academy of Sciences, University of Science and Technology of China, Hefei 230026, China; jiangz10@mail.ustc.edu.cn (Z.J.); dengjie@mail.ustc.edu.cn (J.D.); wdchen@ustc.edu.cn (W.C.); wangdj@ustc.edu.cn (D.W.)

**Keywords:** microwave staring correlated imaging, unsteady aerostat platform, motion parameter fitting, position error

## Abstract

Microwave staring correlated imaging (MSCI), with the technical capability of high-resolution imaging on relatively stationary targets, is a promising approach for remote sensing. For the purpose of continuous observation of a fixed key area, a tethered floating aerostat is often used as the carrying platform for MSCI radar system; however, its non-cooperative random motion of the platform caused by winds and its unbalance will result in blurred imaging, and even in imaging failure. This paper presents a method that takes into account the instabilities of the platform, combined with an adaptive variable suspension (AVS) and a position and orientation system (POS), which can automatically control the antenna beam orientation to the target area and measure dynamically the position and attitude of the stochastic radiation radar array, respectively. By analyzing the motion feature of aerostat platform, the motion model of the radar array is established, then its real-time position vector and attitude angles of each antenna can be represented; meanwhile the selection matrix of beam coverage is introduced to indicate the dynamic illumination of the radar antenna beam in the overall imaging area. Due to the low-speed discrete POS data, a curve-fitting algorithm can be used to estimate its accurate position vector and attitude of each antenna at each high-speed sampling time during the imaging period. Finally, the MSCI model based on the unsteady aerostat platform is set up. In the simulations, the proposed scheme is validated such that under the influence of different unstable platform movements, a better imaging performance can be achieved compared with the conventional MSCI method.

## 1. Introduction

Microwave remote sensing has the ability to work in all day and all weather conditions [1], thus it has been used in many civilian and military fields, such as disaster monitoring and military reconnaissance [2]. The conventional high-resolution microwave remote sensing commonly applies Synthetic Aperture Radar (SAR) which is based on Range-Doppler (RD) principle [3]. However relative motion between radar and target is necessary for SAR and the revisit period is long. In forward-looking or staring imaging geometry, SAR cannot work effectively and encounters great challenges to obtain high-resolution imaging.

Microwave staring correlated imaging is a novel high-resolution staring imaging technique without the relative motion limit of target [4,5,6]. The essence of MSCI is to construct temporal-spatial stochastic radiation field (TSSRF) in the imaging region, which is typically realized by a multi-transmitters configuration emitting independent stochastic waveforms [7,8]. By correlation process (CP) between the target scattering echo and the TSSRF, targets within the antenna beam can be resolved. Due to its superior imaging performance without target relative motion, MSCI has attracted increasing attention and made progress in many aspects such as random radiation source optimization [9,10,11], imaging algorithm [12,13,14] and outfield imaging experiment [15].

At present, research on MSCI depends on the premise of an ideal stable imaging platform, i.e., the system platform of the MSCI radar is assumed to be stationary. However, it is not guaranteed in practical applications. To observe a fixed area, the MSCI radar needs to be raised to a certain height. A tethered aerostat is suitable to serve as the platform of MSCI radar with advantages of long-stay time in the air, wide coverage area and low cost [16,17], but it cannot keep absolutely stationary in the air because of the non-cooperative motion caused by wind and unbalance. The platform instability will result in imaging system errors and the imaging performance will be seriously degraded when the random motion of platform becomes intense.

The imaging system errors in MSCI have been investigated by many studies, since it generally exists in practice. For example, to compensate the gain–phase error in MSCI, Zhou et al. propose a sparse auto-calibration method, which is a cyclic iteration processing combined target reconstruction with gain–phase error estimation [18]. In reference [19], the MSCI with phase error is formulated as a Bayesian hierarchical prior modeling, and self-calibration variational message passing (SC-VMP) algorithm is proposed, which estimates the scattering coefficient and phase error iteratively by VMP and Newton’s method to improve the performance of MSCI with phase error. To estimate the gain–phase error and the synchronization error under high SNR, Tian et al. add a reference receiver to the MSCI system to receive the direct wave signal and the gain–phase error and the synchronization error are estimated by the direct wave signals [20]. In reference [21], a method of strip-mode MSCI with self-calibration of gain–phase errors is proposed to solve the problem of MSCI with gain–phase errors in a large scene. Reference [22] considers the off-grid problem in MSCI and an algorithm based on variational sparse Bayesian learning (VSBL) is developed to solve the MSCI with off-grid problem. Reference [23] focuses on sparsity-driven MSCI with array position error (APE) and propose two sparse auto-calibration imaging algorithms in sparse Bayesian learning framework to compensate the APE. Li et al. analyzes the target-motion-induced error and provides an applicable approach for MSCI in the presence of target-motion-induced error [24]. Hitherto, research on MSCI system error generally concentrated on gain–phase error, off-grid error, APE, and target-motion-induced error. There is no study on the imaging system error caused by instability of the platform which is an important issue in practice applications.

Aiming at the above problems, this paper proposes a MSCI method based on unsteady aerostat platform. In the proposed method, the antenna array with multiple transmitters and one receiver is mounted on the aerostat platform combined with an adaptive variable suspension (AVS), and the position and orientation system (POS) located at the center of the array, controlling its antenna beam orientation to the target area and measuring dynamically its position and attitude during imaging process. The effects of antenna motion and dynamic beam coverage caused by instability of the platform are considered in imaging model to reduce the imaging model error. For antenna motion, the real-time position vectors of antenna are used in imaging model in place of static position vector. The calculation of real-time position vector of antenna depends on the translational speed and the rotational angular velocity of the array in each signal pulse, then based on the low-speed discrete POS data, a least square curve-fitting method is employed to estimate the accurate translational speed and rotational angular velocity of the array at every sampling time. For dynamic beam coverage, the selection matrix of beam coverage calculated by the position and the attitude of the array is introduced to indicate the illuminated area at each pulse.

The rest of this paper is organized as follows. Section 2 presents the MSCI method based on unsteady aerostat platform. In Section 3, estimation of translational speed and rotational angular velocity of antenna array is given. In Section 4, serval simulations are demonstrated to show the effectiveness of the proposed method. Section 5 concludes this paper.

## 2. MSCI Method Based on Unsteady Aerostat Platform

### 2.1. Imaging Scene

**Figure 1:**
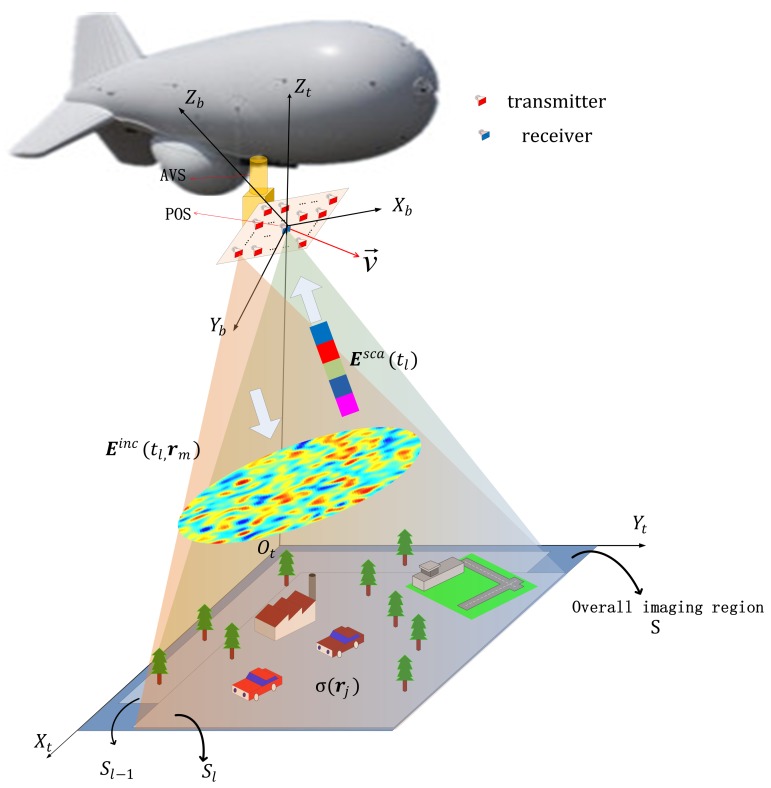
Imaging geometry of MSCI based on unsteady aerostat platform.

MSCI can be realized by using a multi-transmitter configuration to transmit time-independent and group-orthogonal waveforms. To realize observation of targets on ground, MSCI radar can be raised to the air by a tethered aerostat. As shown in Figure 1, the antenna array with *N* transmitters and one receiver at its array center is carried by AVS which is able to control the antenna beam orientation, and POS is placed at the center of the array to dynamically measure its position and the attitude during the imaging process.

To illustrate the geometry of the imaging scene, as the earth-surface inertial reference frame, the coordinate system $O_{t}X_{t}Y_{t}Z_{t}$ is established, with its origin $O_{t}$ located in the projection point of the array center on the ground on $O_{t}X_{t}Y_{t}$ plane at the beginning imaging time, its $X_{t}$ axis pointing to the east along local latitude line, its $Y_{t}$ axis pointing to the north along local meridian and its $Z_{t}$ axis pointing upward along the local geographic vertical line.

The independent signal of random frequency hopping transmitted synchronously by all transmitters and the signal transmitted by the *n*-th transmitter is denoted as

(1) $$s_{n}\left(t\right)=\sum_{l=1}^{L}rect\left[\frac{t-\left(l-1\right)T_{p}}{T}\right]\exp\left\{j2\pi f_{nl}\left[t-\left(l-1\right)T_{p}\right]\right\},$$

where $f_{nl}$ is the frequency of the *l*-th pulse emitted by the *n*-th transmitter and randomly selected within the system bandwidth. rect(t) is rectangular function. *L* is the total number of pulses. $T_{P}$ denotes pulse repetition interval and *T* is pulse width.

**Figure 2:**
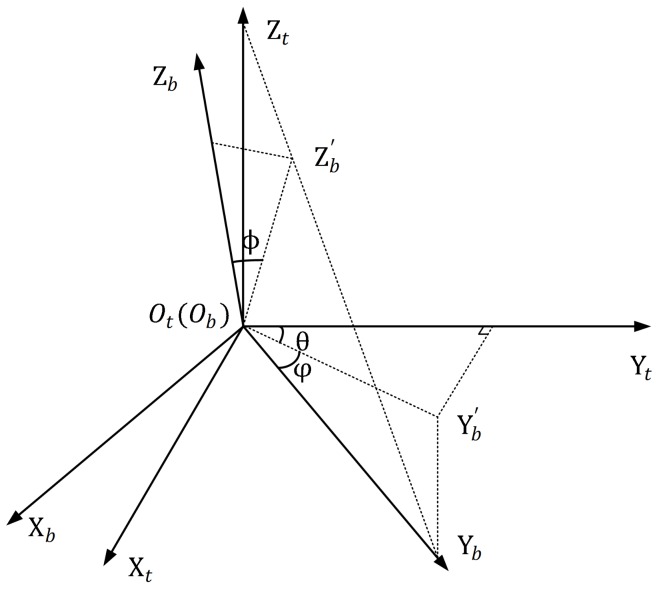
Graphical diagram for the altitude angles.

During the imaging process, POS will dynamically record the position and the attitude of the antenna array. The attitude of the array Euler angles includes yaw angle, pitch angle, and roll angle. To give definition of these angles, the aerostat coordinate system $O_{b}X_{b}Y_{b}Z_{b}$ is established on the array with its origin $O_{b}$ located at its array center, its $X_{b}$ axis pointing to the right along the horizontal axis of the array, its $Y_{b}$ axis pointing forward along the longitudinal axis of the array and its $Z_{b}$ axis perpendicular to the radar array plane. The yaw angle θ is defined as the angle between the projection of $Y_{b}$ on the $O_{t}X_{t}Y_{t}$ plane and the $Y_{t}$ axis, with the $Y_{b}$ axis right side being positive. The pitch angle φ is defined as the angle between the $Y_{b}$ axis and the $O_{t}X_{t}Y_{t}$ plane, with $Y_{b}$ axis up side being positive. The roll angle ϕ is defined as the angle between the $Z_{b}$ axis and the vertical plane containing the $Y_{b}$ axis, with $Z_{b}$ axis right side being positive. The graphical diagram for the altitude angles is shown in Figure 2. ${Y^{\prime }}_{b}$ is the projection of $Y_{b}$ on the $O_{t}X_{t}Y_{t}$ plane and ${Z^{\prime }}_{b}$ is the projection of $Z_{b}$ on the $O_{t}Z_{t}Y_{b}$ plane.

### 2.2. Real-Time Position Vector of Antenna

To eliminate the influence of antenna motion, the real-time position vector $r^{n}\left(t_{l}\right)$ and $r^{s}\left(t_{l}\right)$ are introduced to the MSCI model based on unsteady aerostat platform, where $r^{n}\left(t_{l}\right)$ and $r^{s}\left(t_{l}\right)$ denote the real-time position vector of the *n*-th transmitter and the receiver at $t_{l}$ in the *l*-th pulse in $O_{t}X_{t}Y_{t}Z_{t}$ respectively.

The complicated motion of the antenna array is decomposed into three-dimensional translations and three rotational components. The three-dimensional translations are along $X_{t}$, $Y_{t}$ and $Z_{t}$ respectively. The rotational components are rotation of yaw angle, pitch angle, and roll angle, respectively. As the pulse repetition interval $T_{P}$ is short, the translational speed and the rotational angular velocity will not change drastically during such a short period, so the assumption on the array motion is made that the antenna array motion is uniform translation and uniform rotation during each pulse repetition interval $T_{P}$. Hence the translational speed of the antenna array during the *l*-th pulse is denoted as $v_{l}=\left[v_{l,x},v_{l,y},v_{l,z}\right]$, where $v_{l,x},v_{l,y},v_{l,z}$ are the speeds of the three-dimensional translations along $X_{t}$ axis, $Y_{t}$ axis and $Z_{t}$ axis respectively. The rotational angular velocity of the antenna array during the *l*-th pulse is denoted as $\omega_{l}=\left[\omega_{l,\theta },\omega_{l,\varphi },\omega_{l,\phi }\right]$, where $\omega_{l,\theta },\omega_{l,\varphi },\omega_{l,\phi }$ are rotational angular velocity of the yaw angle, the pitch angle, and the roll angle respectively.

**Figure 3:**
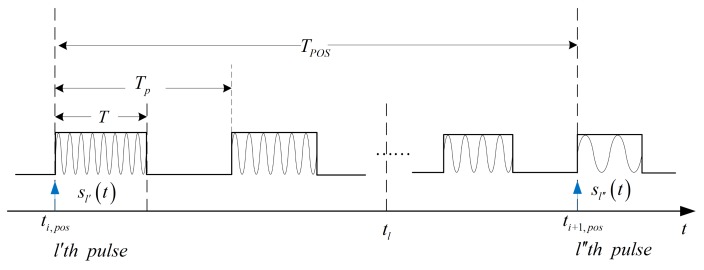
Pulse and POS data timing diagram.

If the motion of antenna array during each pulse is known, the real-time position vector of the antenna can be determined. $T_{pos}$ denotes the repetition period of POS recording data. As Figure 3 shows, the pulse repetition interval $T_{P}$ is far shorter than $T_{pos}$ of POS, so there are many transmitting pulses between adjacent POS data. Assuming that the recorded time $t_{i,pos}$ of the *i*-th POS data is in the $l^{\prime }$-th pulse and the recorded time $t_{i+1,pos}$ of the next POS data is in the $l^{\prime \prime }$-th pulse, the real-time position vector of *n*-th transmitter $r^{n}\left(t_{l}\right)$ at $t_{l}$ in the *l*-th $\left(l^{\prime }\leq l\leq l^{\prime \prime }\right)$ pulse can be expressed as

(2) $$r^{n}\left(t_{l}\right)=r^{n}\left(t_{i,pos}\right)+\Delta r_{v}\left(t_{l}-t_{i,pos}\right)+\Delta r_{\omega }^{n}\left(t_{l}-t_{i,pos}\right),$$

where $\Delta r_{v}\left(t_{l}-t_{i,pos}\right)$ and $\Delta r_{\omega }^{n}\left(t_{l}-t_{i,pos}\right)$ are the displacement vectors of the *n*-th transmitter caused by translation and rotation during $t_{l}-t_{i,pos}$, respectively. $r^{n}\left(t_{i,pos}\right)$ is the position vector of the *n*-th transmitter at $t_{i,pos}$ and can be calculated by the following formula

(3) $$r^{n}\left(t_{i,pos}\right)=r^{s}\left(t_{i,pos}\right)+C\left(\theta_{t_{i,pos}},\varphi_{t_{i,pos}},\phi_{t_{i,pos}}\right)r_{b}^{n},$$

where $r_{b}^{n}$ is the position vector of the *n*-th transmitter in $O_{b}X_{b}Y_{b}Z_{b}$. $r^{s}\left(t_{i,pos}\right)$ is the position vector of the receiver measured by the POS at $t_{i,pos}$ in $O_{t}X_{t}Y_{t}Z_{t}$. $C\left(\theta_{t_{i,pos}},\varphi_{t_{i,pos}},\phi_{t_{i,pos}}\right)$ is the Direction Cosine Matrix (DCM) that transforms the coordinate from $O_{b}X_{b}Y_{b}Z_{b}$ to $O_{t}X_{t}Y_{t}Z_{t}$. The DCM can be expressed as

(4) $$\begin{array}{ll} C\left(\theta_{t_{i,pos}},\varphi_{t_{i,pos}},\phi_{t_{i,pos}}\right)= & \left[\begin{array}{ccc} \cos\left(\theta_{t_{i,pos}}\right) & \sin\left(\theta_{t_{i,pos}}\right) & 0\\ -\sin\left(\theta_{t_{i,pos}}\right) & \cos\left(\theta_{t_{i,pos}}\right) & 0\\ 0 & 0 & 1 \end{array}\right]\times \\ & \left[\begin{array}{ccc} 1 & 0 & 0\\ 0 & \cos\left(\varphi_{t_{i,pos}}\right) & -\sin\left(\varphi_{t_{i,pos}}\right)\\ 0 & \sin\left(\varphi_{t_{i,pos}}\right) & \cos\left(\varphi_{t_{i,pos}}\right) \end{array}\right]\times \left[\begin{array}{ccc} \cos\left(\phi_{t_{i,pos}}\right) & 0 & \sin\left(\phi_{t_{i,pos}}\right)\\ 0 & 1 & 0\\ -\sin\left(\phi_{t_{i,pos}}\right) & 0 & \cos\left(\phi_{t_{i,pos}}\right) \end{array}\right] \end{array}.$$

As the receiver is at the center of the antenna array, its position vector at $t_{l}$ is only affected by the translation of the antenna array during the period of $t_{l}-t_{i,pos}$ and can expressed as

(5) $$r^{s}\left(t_{l}\right)=r^{s}\left(t_{i,pos}\right)+\Delta r_{v}\left(t_{l}-t_{i,pos}\right).$$

$\Delta r_{v}\left(t_{l}-t_{i,pos}\right)$ and $\Delta r_{\omega }^{n}\left(t_{l}-t_{i,pos}\right)$ can be calculated by the translational speed and the rotational angular velocity of the antenna array:

(6) $$\Delta r_{v}\left(t_{l}-t_{i,pos}\right)=\left[\min\left\{t_{l},l^{\prime }T_{p}\right\}-t_{i,pos}\right]v_{l^{\prime }}+\sum_{k=l^{\prime }+1}^{l}\left[\min\left\{t_{l},kT_{p}\right\}-\left(k-1\right)T_{P}\right]v_{k},$$

(7) $$\Delta r_{\omega }^{n}\left(t_{l}-t_{i,pos}\right)=C\left(\Delta \theta_{t_{l}},\Delta \varphi_{t_{l}},\Delta \phi_{t_{l}}\right)r_{b}^{n}-r_{b}^{n}.$$

The function min{x,y} returns the minimum of *x* and *y*. $\Delta \theta_{t_{l}}$, $\Delta \varphi_{t_{l}}$ and $\Delta \phi_{t_{l}}$ are the changes of the altitude angles during $t_{l}-t_{i,pos}$ and can be calculated by the following formula

(8) $$\alpha_{t_{l}}=\left[\min\left\{t_{l},l^{\prime }T_{p}\right\}-t_{i,pos}\right]\omega_{l^{\prime },\alpha }+\sum_{k=l^{\prime }+1}^{l}\left[\min\left\{t_{l},kT_{p}\right\}-\left(k-1\right)T_{P}\right]\omega_{k,\alpha },$$

where $\alpha_{t_{l}}\in \left(\Delta \theta_{t_{l}},\Delta \varphi_{t_{l}},\Delta \phi_{t_{l}}\right)$.

### 2.3. Influence of Platform Motion on Beam Coverage

The aerostat platform instability not only causes the antenna motion, but also changes the beam coverage in the overall imaging region *S*. All echo data contains the information of all beam covered areas, therefore as the union of all beam coverages, the overall imaging region *S* is considered in imaging. The selection matrix of beam coverage is introduced to indicate the dynamically illuminated area of each pulse within the overall imaging region.

The beam coverage of the *l*-th pulse is denoted as $S_{l}$, and the coordinate $\left(x_{l}^{c},y_{l}^{c}\right)$ is the beam coverage center on the $O_{t}X_{t}Y_{t}$ plane of the *l*-th pulse:

(9) $$x_{l}^{c}=x_{l}^{s}-\left(tan\phi_{l}cos\varphi_{l}/cos\theta_{l}-sin\varphi_{l}tan\theta_{l}\right)z_{l}^{s},$$

(10) $$y_{l}^{c}=y_{l}^{s}+\left(tan\phi_{l}sin\varphi_{l}/cos\theta_{l}+cos\varphi_{l}tan\theta_{l}\right)z_{l}^{s},$$

where $\left(x_{l}^{s},y_{l}^{s},z_{l}^{s}\right)$, $\left(\theta_{l},\varphi_{l},\phi_{l}\right)$ are its center position coordinate and its attitude angles of the antenna array at the start time of the *l*-th pulse.

The overall imaging region *S* is the union of all beam covered areas during imaging, i.e., $S=S_{1}⋃S_{2}⋃\ldots ⋃S_{L}$. The size of the beam covered area of a single pulse is denoted as $w_{x}\times w_{y}$, where $w_{x},w_{y}$ are the side length. The size of the overall imaging region is

(11) $$W_{x}\times W_{y}=\left(x_{max}^{c}-x_{min}^{c}+w_{x}\right)\times \left(y_{max}^{c}-y_{min}^{c}+w_{y}\right),$$

where $W_{x}$, $W_{y}$ are the side length of *S*. $x_{\max}^{c}$ and $x_{min}^{c}$ are the maximum value and the minimum value of $x_{l}^{c}$, l=1,2,⋯,L. $y_{\max}^{c}$ and $y_{min}^{c}$ are the maximum value and the minimum value of $y_{l}^{c}$, l=1,2,⋯,L.

The overall imaging region will be discretized into M=P×Q discrete grids, where *P* is the row number of azimuth resolution cells, and *Q* is the column number of range resolution cells. In $O_{t}X_{t}Y_{t}Z_{t}$, the position vectors of the *m*-th grid is denoted as $r_{m}$, m=1,2,⋯,M.

Selection matrix of beam coverage is as below

(12) $$D=\left[\begin{array}{cccc} D_{1}\left(1\right) & D_{1}\left(2\right) & \cdots & D_{1}\left(M\right)\\ D_{2}\left(1\right) & D_{2}\left(2\right) & \cdots & D_{2}\left(M\right)\\ \vdots & \vdots & \vdots & \vdots \\ D_{L}\left(1\right) & D_{L}\left(2\right) & \cdots & D_{L}\left(M\right) \end{array}\right].$$

The element $D_{l}\left(m\right)$ indicates whether the *m*-th grid is illuminated by the *l*-th pulse beam:

(13) $$D_{l}\left(m\right)=\left\{\begin{array}{c} 1\;if\;\left(r_{m}\in S_{l}\right)\\ 0\;if\;\left(r_{m}\notin S_{l}\right) \end{array}\right.$$

### 2.4. Imaging Equation

Since the whole imaging region *S* has been divided into M=P×Q discrete grids. The scattering coefficient of the *m*-th grid is $\sigma (r_{m})$. At the beginning of the *l*-th pulse, each transmitter simultaneously transmits independent and stochastic signal. All signals are superimposed in *S* to generate TSSRF. The radiation field at $r_{m}$ can be expressed as

(14) $$E^{inc}\left(t_{l},r_{m}\right)=\sum_{n=1}^{N}D_{l}\left(m\right)\frac{F_{n}\left({\hat{R}}_{n}\right)s_{n}\left(t_{l}-\left|r_{m}-r^{n}\left(t_{l,0}\right)\right|/c\;\right)}{4\pi \left|r_{m}-r^{n}\left(t_{l,0}\right)\right|},$$

where ${\hat{R}}_{n}=\left[r_{m}-r^{n}\left(t_{l,0}\right)\right]/\left|r_{m}-r^{n}\left(t_{l,0}\right)\right|$. $F_{n}\left({\hat{R}}_{n}\right)$ denotes the radiation pattern of the *n*-th transmitter antenna. $t_{l,0}=\left(l-1\right)T$ denotes the initial time of the *l*-th pulse.

The radiation field interacts with the targets and the received echo can be expressed as

(15) $$E^{sca}\left(t_{l}\right)=\sum_{m=1}^{M}\sigma \left(r_{m}\right)\frac{E^{inc}\left(t_{l}-\frac{\left|r^{s}\left(t_{l}\right)-r_{m}\right|}{c},r_{m}\right)}{4\pi \left|r^{s}\left(t_{l}\right)-r_{m}\right|}F_{s}\left({\hat{R}}_{s}\right)+n\left(t_{l}\right),$$

where ${\hat{R}}_{s}=\left[r_{m}-r^{s}\left(t_{l}\right)\right]/\left|r_{m}-r^{s}\left(t_{l}\right)\right|$. $F_{s}\left({\hat{R}}_{s}\right)$ denotes the radiation pattern of the receiver antenna. $n\left(t_{l}\right)$ denotes the additive noise.

Considering the round-trip propagation of the electromagnetic field in the free space, the modified radiation field is defined as

(16) $$E^{rad}\left(t_{l},r_{m}\right)=\sum_{n=1}^{N}\left\{\frac{F_{s}\left({\hat{R}}_{s}\right)F_{n}\left({\hat{R}}_{n}\right)s_{n}\left[t_{l}-\left(|r_{m}-r_{}^{n}\left(t_{l,0}\right)\left|+\right|r_{}^{s}\left(t_{l}\right)-r_{m}|\right)/c\;\right]}{{\left(4\pi \right)}^{2}|r_{m}-r_{}^{n}\left(t_{l,0}\right)\left|\right|r_{}^{s}\left(t_{l}\right)-r_{m}|}D_{l}\left(m\right)\right\}.$$

Let $t_{l,k}$, l=1,2,⋯,L be the sampling time in the *l*-th pulse, thus the imaging equation in the matrix vector form can be written as

(17) $$E^{sca}=E^{rad}\cdot \sigma +n,$$

where $E^{sca}={\left[E^{sca}\left(t_{1,k}\right),E^{sca}\left(t_{2,k}\right),\cdots ,E^{sca}\left(t_{L,k}\right)\right]}^{T}$ is the echo vector, $\sigma ={\left[\sigma \left(r_{1}\right),\sigma \left(r_{2}\right),\cdots ,\sigma \left(r_{M}\right)\right]}^{T}$ is the scattering coefficient vector, $n={\left[n\left(t_{1,k}\right),n\left(t_{2,k}\right),\cdots n\left(t_{L,k}\right)\right]}^{T}$ is the noise vector, $E^{rad}$ is the modified radiation field matrix with ${\left[E^{rad}\right]}_{lm}=E^{rad}\left(t_{l,k},r_{m}\right)$.

The scattering coefficient vector σ can be reconstructed by the correlated processing between $E^{sca}$ and $E^{rad}$, which can be described as

(18) $$\hat{\sigma }=\zeta \left[E^{rad},E^{sca}\right],$$

where ζ denote the correlated operator.

Common correlated imaging algorithms include Pseudo-Inverse algorithm, Tikhonov regularization, TV regularization, and sparse reconstruction algorithms, such as Orthogonal Matching Pursuit, sparse Bayesian learning, etc. This paper adopts Tikhonov regularization algorithm because it is robust to noise and does not require a priori of the target. Tikhonov regularization can be formulated as the following optimization problem

(19) $$\hat{\sigma }=\underset{\sigma }{\arg\min}\;\left\{∥E^{sca}-E^{rad}\cdot \sigma ∥+\lambda {∥\sigma ∥}_{2}^{2}\right\},$$

where λ is the regularization parameter.

## 3. Estimation of Translational Speed and Rotational Angular Velocity

POS system sets inertial navigation technology and satellite navigation technology in one body, and adopts the real-time and post-process information fusion respectively to get high precision positioning and orientation information. For MSCI, the imaging time is very short, so the error accumulation of the INS is negligible, and the INS is more accurate in a short time. Hence the measured position and angular data for estimation of translational speed and rotational angular velocity are very accurate.

The calculation of the real-time position vector of each antenna at high-speed sampling time requires the translational speed and the rotational angular velocity of the antenna array. The low-speed discrete POS data will be used to estimate the translational speed and the rotational angular velocity. Since the data rate of POS is usually less than the pulse repetition frequency as Figure 3 shows, there are many pulses between two adjacent POS data. To obtain the translational speed and the rotational angular velocity of the antenna array during each pulse, third-order polynomial curve fitting to the position and attitude is employed and the least squares method is used to obtain the coefficients of the fitting polynomial. The fitting polynomial of the position or the attitude can be expressed as

(20) $$\mu \left(t\right)=\sum_{k=0}^{3}a_{\mu ,k}t^{k},$$

where $a_{\mu ,k}$ is the coefficient of the polynomial, and $\mu \left(t\right)$ is the fitting curve.

The initial time of the *l*-th pulse is denoted as $t_{l,0}$, and the end time of the *l*-th pulse is denoted as $t_{l+1,0}=t_{l,0}+T_{p}$. By substituting $t_{l,0}$ and $t_{l+1,0}$ into the fitting curve $\mu \left(t\right)$, we can get the position or the attitude parameters at the beginning and the end of each pulse. Based on the assumption that the antenna array is uniformly translated and rotated during each pulse, the translational speed and rotational angular velocity in each pulse can be solved by

(21) $$\omega_{l}=\left[\begin{array}{c} \frac{\theta \left(t_{l+1,0}\right)-\theta \left(t_{l,0}\right)}{T}\\ \frac{\varphi \left(t_{l+1,0}\right)-\varphi \left(t_{l,0}\right)}{T}\\ \frac{\phi \left(t_{l+1,0}\right)-\phi \left(t_{l,0}\right)}{T} \end{array}\right],$$

(22) $$v_{l}=\frac{r^{s}\left(t_{l+1,0}\right)-r^{s}\left(t_{l,0}\right)}{T}.$$

## 4. Simulation

**Figure 4:**
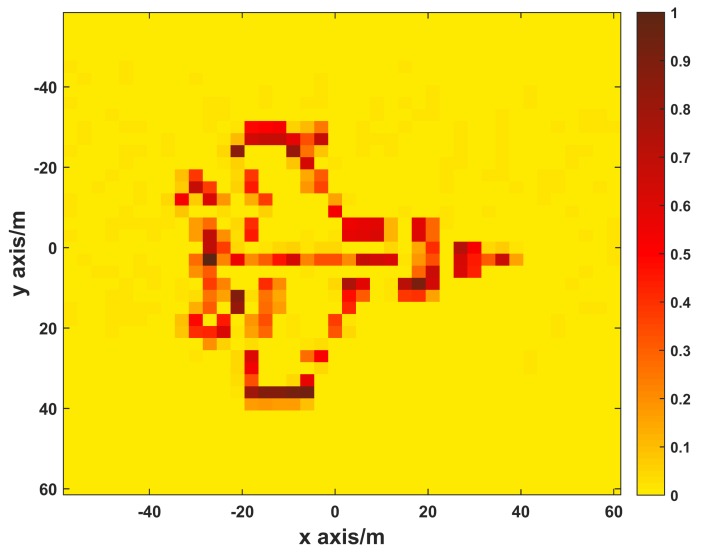
Target image.

In this section, simulations are demonstrated to verify the proposed method based on unsteady aerostat platform. The scenario for the simulation is shown in Figure 1. An X-band MSCI radar system with carrier frequency of 10 GHz is considered. The randomly radiating radar array with 25 transmitters and 1 receiver is raised to 350 m height by a tethered aerostat. The main system simulation parameters are given in Table 1, and the target model is shown in Figure 4. In simulations, the measurement errors of position and altitude angles are assumed to be independent and subject to Gauss distribution with zero mean and 1 mm standard deviation for position and $0.05^{{}^{\circ}}$ standard deviation for altitude angles.

**Figure 5:**
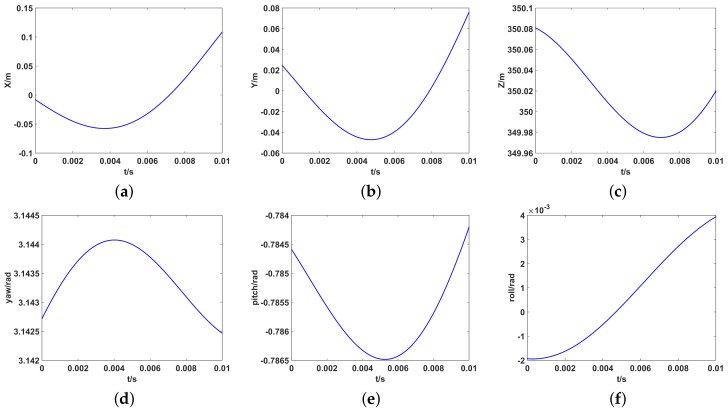
The motion trajectory of each component. (**a**) the translational component along the $X_{t}$; (**b**) the translational component along the $Y_{t}$; (**c**) the translational component along the $Z_{t}$; (**d**) the rotation of yaw; (**e**) the rotation of pitch; (**f**) the rotation of roll.

To illustrate the effectiveness of the proposed method, the trajectories in Figure 5 are used as the three-dimensional translations and the rotational components of the antenna array caused by unsteady platform.

### 4.1. Verification of the Proposed Model

In this subsection, simulations are taken to compare the imaging performance of different imaging models. The proposed imaging model based on unsteady aerostat platform (UPIM) will be compared with the imaging model for stationary platform (SPIM) and imaging model which only uses the discrete POS data (DPDIM). SPIM ignores the motion of antenna array and assumes that the position vector of antenna and the beam coverage do not change during the imaging process. When calculating the radiation field, SPIM uses the first recorded POS data as the position and the attitude angles of the array, i.e., $r^{n}\left(t_{l}\right)=r^{n}\left(t_{1,pos}\right)\left(l=1,2,\cdots ,L\right)$. DPDIM does not fit the discrete POS data and uses the closest POS data for each pulse. The normalized mean square error (NMSE) is used to quantity the reconstruction performance, with the definition as: $NMSE={{∥\hat{x}-x∥}_{2}/∥x∥\;}_{2}$, where $\hat{x}$ and x denote the reconstructed and true value of target.

**Figure 6:**
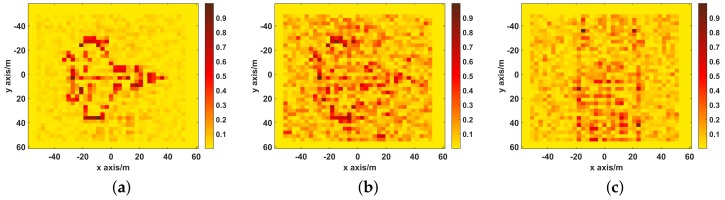
The imaging results of UPIM, DPDIM, and SPIM. (**a**) the reconstructed image by UPIM, the NMSE is 0.28; (**b**) the reconstructed image by DPDIM, the NMSE is 0.94; (**c**) the reconstructed image by SPIM, the NMSE is 1.21.

The imaging results are depicted in Figure 6. As shown in Figure 6a, Comparably, the image reconstructed by UPIM is focused with quite a few spurious scatters, whose better imaging performance benefits from the fact that the UPIM has the minimal motion estimation error. In Figure 6b, apart from strong scatters, the image reconstructed by DPDIM has many spurious scatters. In Figure 6c, the reconstructed image by SPIM is defocused and blurry, and the target is hard to recognize.

**Figure 7:**
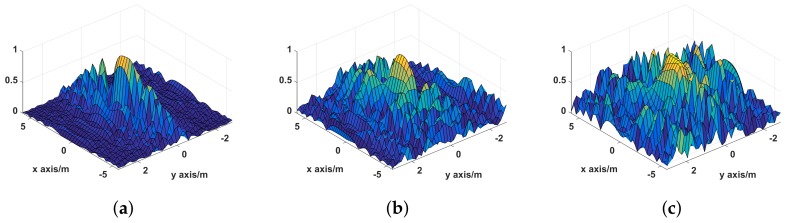
The point spread function of UPIM, DPIM, and SPIM. (**a**) UPIM; (**b**) DPIM; (**c**) SPIM.

**Figure 8:**
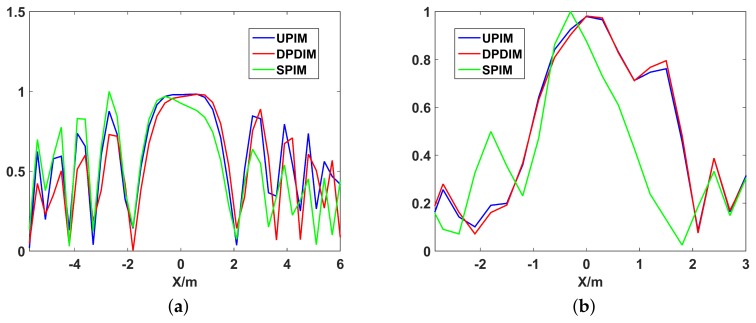
The profile of the point spread function of UPIM, DPIM, and SPIM. (**a**) the *X*-axis profile of the point spread function; (**b**) the *Y*-axis profile of the point spread function.

The point spread functions (PSF) of UPIM, DPIM, and SPIM are illustrated in Figure 7 and the *X*-axis and *Y*-axis profiles of the PSF are shown in Figure 8. It can be seen from Figure 7, the number and the level of the side lobes is minimum for UPIM while the other two methods both have more side lobes and higher level of side lobes. Figure 8 shows that these methods have almost the same width of the main lobe in *X*-axis profile and the *Y*-axis profile. The above simulation results demonstrate that UPIM indeed reduces the number and the level of side lobes caused by platform instability, but it does not improve the imaging resolution of MSCI.

**Figure 9:**
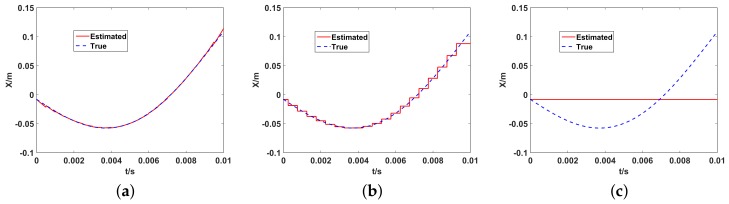
The estimated and the real trajectory of the translation along the $X_{t}$. (**a**) UPIM; (**b**) DPDIM; (**c**) SPIM.

For the three imaging models mentioned above, Figure 9 shows the fitting effect on the translational motion trajectory of the aerostat platform along the $X_{t}$. It can be seen that the proposed UPIM model has the best fitting effect that the estimated translational trajectory is almost the same with the real one with the most minimum motion estimation error, which obviously benefits a better imaging performance in UPIM.

**Figure 10:**
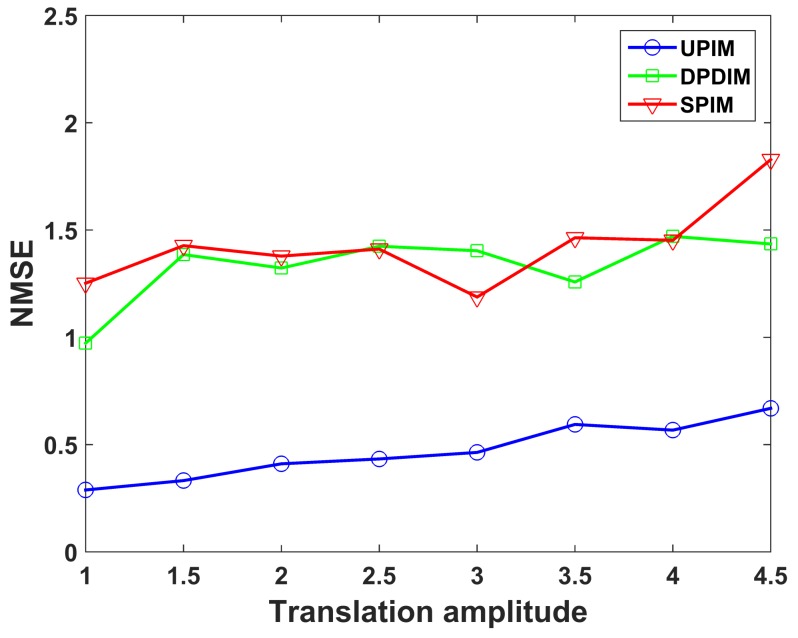
NMSE of the imaging results by UPIM, DPDIM, and SPIM at different amplitudes of translation.

To verify the effectiveness of the proposed method with the translation amplitude increasing, the relationship between the imaging quality and the translation amplitude for three imaging models is presented in Figure 10. The amplitude of three-dimensional translations gradually increased by the step of 0.5 times the original amplitude shown in Figure 5, while the rotation amplitudes keep constant. The coordinate of the horizontal axis in Figure 10 represents the multiple of the original translation amplitude. As seen from Figure 10, the imaging performance of UPIM is still better than the other imaging models when the amplitude of translation increases.

**Figure 11:**
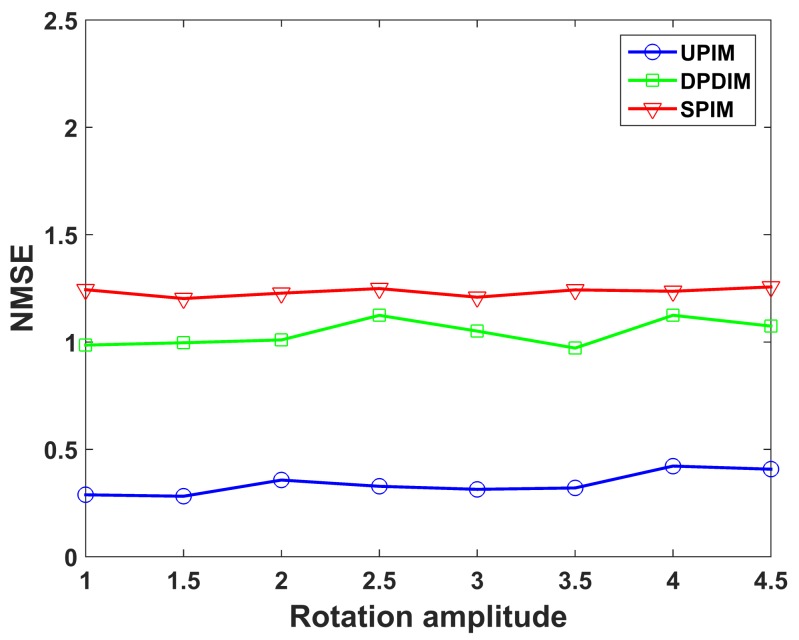
NMSE of the imaging results by UPIM, DPDIM, and SPIM at different amplitudes of rotation.

The imaging quality under different rotation amplitudes is depicted in Figure 11. The amplitude of all rotational components is gradually increased by step of 0.5 times the original rotational amplitude, while the translation amplitudes keep constant. Figure 11 shows that the proposed method has better performance under all rotation amplitudes.

### 4.2. Effect of Different Translational Components on Imaging Performance

**Figure 12:**
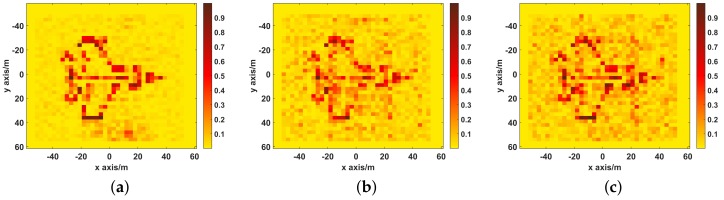
The imaging results of UPIM when only one translation component exists. (**a**) Only translational component along the $X_{t}$ exists, the NMSE is 0.21; (**b**) Only translational component along the $Y_{t}$ exists, the NMSE is 0.58; (**c**) Only translational component along the $Z_{t}$ exists, the NMSE is 0.59.

This section is to study the effect of independent translational component on imaging performance. In simulations, all independent translational components use the same motion trajectory as shown in Figure 5a. Figure 12 shows the imaging results reconstructed by UPIM when only one translational component exists.

**Figure 13:**
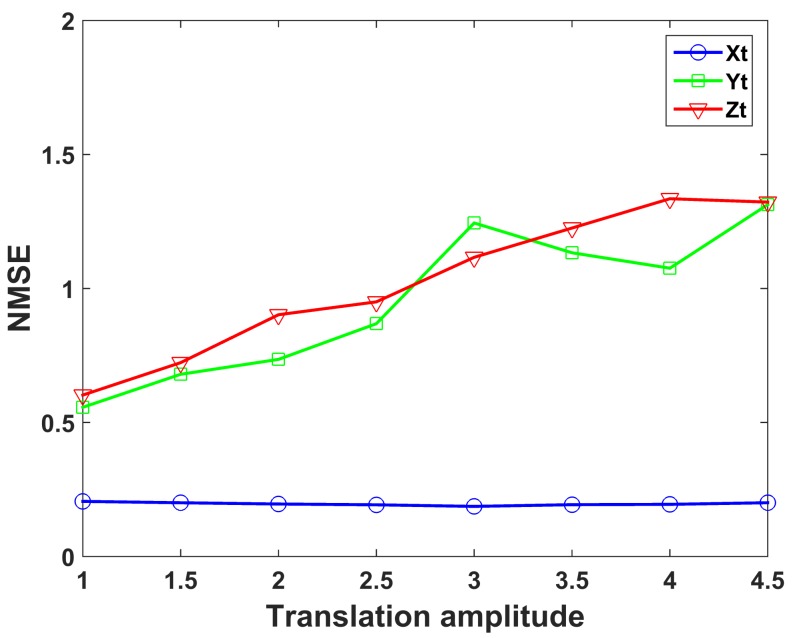
NMSE of the imaging results when only one translational component exists at different amplitudes.

The imaging quality for each translational component under different translation amplitudes is presented in Figure 13. As shown in Figure 12 and Figure 13, the translation along the $X_{t}$ has the minimal influence on imaging performance, while both translation along the $Y_{t}$ and $Z_{t}$ has almost the same influence on imaging performance. Therefore, for improving the image performance, the position of the antenna array along the $Y_{t}$ and the $Z_{t}$ should be estimated more accurate.

**Figure 14:**
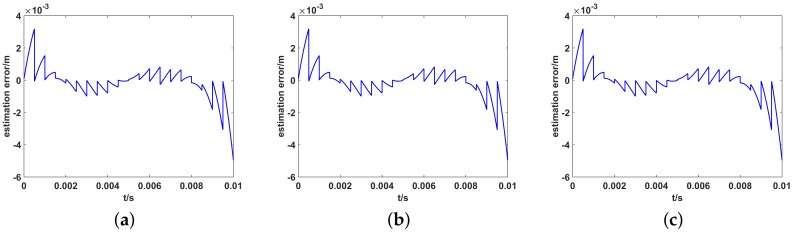
The estimation error of antenna position in three-dimensional translations. (**a**) Only along the $X_{t}$; (**b**) Only along the $Y_{t}$; (**c**) Only along the $Z_{t}$.

Next, we investigate the reason for the different imaging results in three-dimensional translations. Although the proposed method compensates in part of the non-cooperative motion, due to the limited number of POS data, the estimated translation errors cannot be totally eliminated. Figure 14 shows the estimation error in three-dimensional translations. The accurate calculation of the radiation field is directly related to the round-trip propagation delay of the electromagnetic wave between antenna and target. The propagation time delay error caused by estimation error of array position will lead to the calculated radiation field error. Because the translational estimation errors in three dimensions have different effect on the propagation time delay, the influence of different translational components on imaging is not the same.

Assuming at *t* time, the coordinate of the *i*-th antenna is $\left(x_{a},y_{a},z_{a}\right)$ and the coordinate of any point *m* in the imaging region is $\left(x,y,z\right)$. After Δt, the coordinate of the antenna become $\left(x_{a}+\Delta x_{a},y_{a}+\Delta y_{a},z_{a}+\Delta z_{a}\right)$.

The distance from the *i*-th antenna to the point *m* in imaging region is

(23) $$S_{im}=\sqrt{{\left(x-x_{a}\right)}^{2}+{\left(y-y_{a}\right)}^{2}+{\left(z-z_{a}\right)}^{2}}.$$

The partial differential of the propagation path along three coordinate dimensions is

(24) $$\frac{\partial S_{im}}{\partial x_{a}}=\frac{x_{a}-x}{\sqrt{{\left(x-x_{a}\right)}^{2}+{\left(y-y_{a}\right)}^{2}+{\left(z-z_{a}\right)}^{2}}},$$

(25) $$\frac{\partial S_{im}}{\partial y_{a}}=\frac{y_{a}-y}{\sqrt{{\left(x-x_{a}\right)}^{2}+{\left(y-y_{a}\right)}^{2}+{\left(z-z_{a}\right)}^{2}}},$$

(26) $$\frac{\partial S_{im}}{\partial z_{a}}=\frac{z_{a}-z}{\sqrt{{\left(x-x_{a}\right)}^{2}+{\left(y-y_{a}\right)}^{2}+{\left(z-z_{a}\right)}^{2}}}.$$

In the simulation scenario, the height of the antenna array is 350 m and the antenna is squint observation with the slanting angle $45^{{}^{\circ}}$. For any point in the imaging area, its coordinate satisfies 291.5≤y≤408.5, −58.5≤x≤58.5 and z=0. Because the size of antenna array is much smaller than the size of imaging region, therefore for most points in the imaging region, it is satisfied that $\left|x\right|\gg \left|x_{a}\right|$ and $\left|y\right|\gg \left|y_{a}\right|$, and the value of partial differential function satisfy that $\left|\partial S_{im}/\partial y_{a}\;\right|>\left|\partial S_{im}/\partial x_{a}\;\right|$. As $z_{a}-z\approx 350$, the partial differential of $S_{im}$ satisfies $\left|\partial S_{im}/\partial z_{a}\;\right|>\left|\partial S_{im}/\partial x_{a}\;\right|$. Therefore the same estimation error along the $X_{t}$ axis will cause less propagation delay error than the other two components, which explains the reason that under the same translational trajectory, the reconstructed image with the translation only along the $X_{t}$ has the best imaging result.

### 4.3. Effect of Different Rotation Components on Imaging Performance

**Figure 15:**
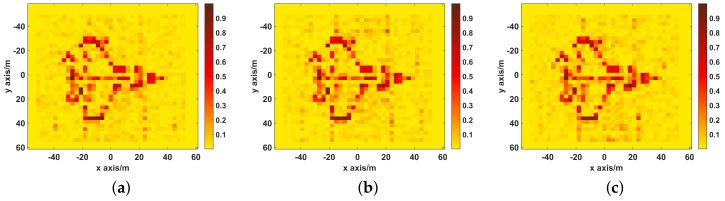
The imaging results of UPIM when only one rotation component exists. (**a**) Only yaw angle, the NMSE is 0.27; (**b**) Only pitch angle, the NMSE is 0.28; (**c**) Only roll angle, the NMSE is 0.28.

**Figure 16:**
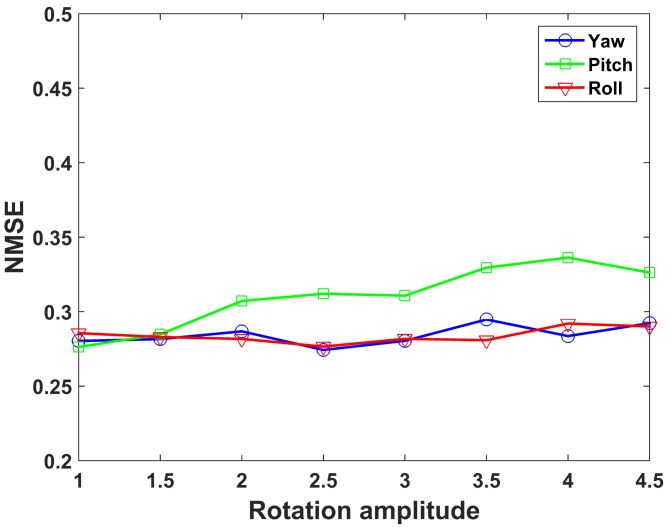
NMSE of the imaging results when only one rotation component exists at different amplitudes.

This section is to study the effect of independent rotational component on imaging performance. In the simulation, three rotational components have the same rotational trajectory as shown in Figure 5d. Figure 15 shows the imaging results reconstructed by UPIM when only one rotational component exists. As three rotational components gradually increased by the step of 0.5 times the original rotational amplitude shown in Figure 5, the imaging quality for each rotational component under different rotation amplitudes is presented in Figure 16.

From Figure 15 and Figure 16, it can be seen that three rotational components have almost the same effect on imaging performance under different rotation amplitudes.

### 4.4. Effect of the Position and Angular-Measuring Accuracy on Imaging Performance

**Figure 17:**
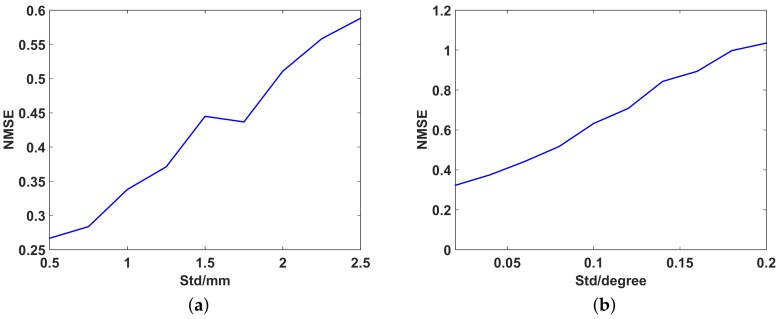
The NMSE of imaging result under different measuring accuracy. (**a**) position-measuring accuracy; (**b**) angular-measuring accuracy.

This section is to study the effect of the measuring accuracy of position and attitude parameters on imaging. The imaging performance under different position accuracy and angular accuracy is simulated. In simulations, the measurement error is assumed to be independent and subject to Gauss distribution with zero mean and different variances. The smaller the variance, the higher the measuring accuracy. Figure 17 shows the imaging quality under different position-measuring accuracy and different angular-measuring accuracy, respectively. The results show that the imaging performance is very sensitive to the measuring accuracy of position and attitude parameters which means that the proposed method has a high demand of accurate measurement of position and attitude parameters.

## 5. Conclusions

In this paper, a novel MSCI method based on unsteady aerostat platform is proposed, where the MSCI radar array is carried by AVS to keep its antenna beam orientation to the target in the non-cooperative motion of the platform caused by the wind etc., and the POS is used to dynamically measure the position and the attitude of the antenna array. By decomposing of the platform motion to its translation and rotation, the motion model of unsteady aerostat platform in air has been built, and for each antenna, its real-time position vector can be calculated by its translational speed and its rotational angular velocity in each pulse, replacing the static position vector in the traditional MSCI model. For the dynamic beam coverage in the whole observation region, a selection matrix of beam coverage is introduced to indicate the illuminated area at each pulse. By analyzing the modified stochastic radiation field and its scattered echo, the MSCI model based on unsteady aerostat platform is established. Furtherly, based on low-speed POS data, a polynomial curve-fitting algorithm is used to eliminate the position error of the radar array. Simulation experiments demonstrate that under its different random translations and rotations of unsteady aerostat platform, the position and attitude of the antenna array at different time can be estimated well, and better imaging performance can be achieved by the proposed scheme, which provides a feasible technical approach for the floating-observation-platform to realize the microwave staring remote sensing observation in the near space.

## Figures and Tables

**Table 1:** Simulation parameter.

Simulation Parameter	Value
Aperture of antenna array	1.5 m × 1.5 m
Number of transmitting antenna	25
Height of array center	350 m
Slanting angle	$\theta_{0}=45^{{}^{\circ}}$
The overall observation area	120 m × 120 m
Beam coverage area by single pulse	105 m × 105 m
Number of grid	40 × 40
Grid spacing	3 m
Signal form	Random frequency hopping
Pulse repetition interval	5 μm
Pulse width	600 ns
Bandwidth	500 MHz
Carrier frequency	10 GHz
Total imaging time	10 ms
